# Population structure and spatio-temporal transmission dynamics of *Plasmodium vivax* after radical cure treatment in a rural village of the Peruvian Amazon

**DOI:** 10.1186/1475-2875-13-8

**Published:** 2014-01-06

**Authors:** Christopher Delgado-Ratto, Veronica E Soto-Calle, Peter Van den Eede, Dionicia Gamboa, Angel Rosas, Emmanuel N Abatih, Hugo Rodriguez Ferrucci, Alejandro Llanos-Cuentas, Jean-Pierre Van Geertruyden, Annette Erhart, Umberto D’Alessandro

**Affiliations:** 1 Unit of International Health, ESOC Department, Faculty of Medicine, University of Antwerp, Universiteitsplein 1, B-2610 Antwerp, Belgium; 2 Laboratory of Malaria, Unit of Molecular Epidemiology, Institute of Tropical Medicine Alexander von Humboldt (IMTAvH)-Universidad Peruana Cayetano Heredia (UPCH), Av Honorio Delgado 430, Lima, Peru; 3 Grupo de Estudio de Leishmaniasis y Malaria (GELM), ITMAvH-Universidad Peruana Cayetano Heredia (UPCH), Av Honorio Delgado 430, Lima 31, Peru; 4 Malariology Unit, Department of Biomedical Sciences, Institute of Tropical Medicine, Antwerp, Nationalestraat 155, B-2000 Antwerp, Belgium; 5 Departamento de Ciencias Celulares y Moleculares, Facultad de Ciencias y Filosofía- Universidad Peruana Cayetano Heredia, Av Honorio Delgado 430, Lima, Peru; 6 Unit of Veterinary Biostatistics and Epidemiology, Institute of Tropical Medicine, Nationalestraat 155, B-2000 Antwerp, Belgium; 7 Ministry of Health, Av 28 de Julio s/n, Punchana, Loreto, Peru; 8 Department of Public Health, Institute of Tropical Medicine, Nationalestraat 155, B-2000 Antwerp, Belgium; 9 Medical Research Council Unit, Fajara PO Box 273, Banjul, The Gambia

**Keywords:** *Plasmodium vivax*, Malaria, Genotyping, Microsatellites, Population genetics, Spatio-temporal analysis, Peruvian amazon

## Abstract

**Background:**

Despite the large burden of *Plasmodium vivax*, little is known about its transmission dynamics. This study explored the population structure and spatio-temporal dynamics of *P. vivax* recurrent infections after radical cure in a two-year cohort study carried out in a rural community of the Peruvian Amazon.

**Methods:**

A total of 37 *P. vivax* participants recruited in San Carlos community (Peru) between April and December 2008 were treated radically with chloroquine and primaquine and followed up monthly for two years with systematic blood sampling. All samples were screened for malaria parasites and subsequently all *P. vivax* infections genotyped using 15 microsatellites. Parasite population structure and dynamics were determined by computing different genetic indices and using spatio-temporal statistics.

**Results:**

After radical cure, 76% of the study participants experienced one or more recurrent *P. vivax* infections, most of them sub-patent and asymptomatic. The parasite population displayed limited genetic diversity (*He* = 0.49) and clonal structure, with most infections (84%) being monoclonal. Spatio-temporal clusters of specific haplotypes were found throughout the study and persistence of highly frequent haplotypes were observed over several months within the same participants/households.

**Conclusions:**

In San Carlos community, *P. vivax* recurrences were commonly observed after radical treatment, and characterized by asymptomatic, sub-patent and clustered infections (within and between individuals from a few neighbouring households). Moreover low genetic diversity as well as parasite inbreeding are likely to define a clonal parasite population which has important implications on the malaria epidemiology of the study area.

## Background

*Plasmodium vivax,* the most widely distributed malaria species, threatens almost 40% of the worldwide population [1]. Vivax malaria is less benign than commonly thought [1] and, due to its complex life cycle, which includes liver hypnozoites that can be re-activated well after the primary infection, it is difficult to have a clear picture of its burden and epidemiology. Limited knowledge of the underlying *P. vivax* population structure and dynamics are major obstacles to achieve effective malaria control, particularly in low endemic areas [2]. In the Peruvian Amazon, vivax malaria is still a major public health problem [3]. Both chloroquine-resistant strains [4] and the frequent occurrence of recurrent infections after ‘radical’ treatment [chloroquine (CQ) and primaquine (PQ)] have been reported [4,5]. Moreover, the frequent occurrence of clustered asymptomatic infections makes malaria control in this low endemic setting extremely difficult [6-8]. Therefore, there is the need to carry out *P. vivax* population genetic studies to understand how parasites are circulating in the human population, as well as related topics such as the spread of drug resistance and the development of clinical immunity [9,10]. Similar to other endemic Amazonian countries [11-13], *P. vivax* populations in the Peruvian Amazon are extremely heterogeneous, with different degrees of diversity and polyclonality [14,15]. This study was part of a larger ongoing *P. vivax* cohort conducted within 21 rural communities, and which results will be published later. In this paper, one of these study villages has been selected in order to analyse, at community and household level, the population structure and the spatial-temporal dynamics of *P. vivax* infections after radical treatment of the primary infection.

## Methods

### Study site

**Figure 1:**
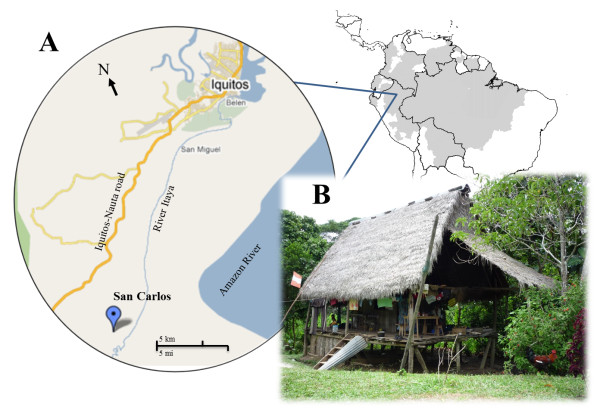
**Map of the study area. A.** Map of the study area, San Carlos community, located at the southeast of Iquitos city, capital of the Peruvian Amazon. **B.** Picture of a typical household in San Carlos community belonging to one *P. vivax*-infected participant (Photography provided by A Erhart).

The study was conducted in San Carlos (latitude 3°57′30.99″S longitude 73°20′45.76″W), a relatively small and isolated village in the Peruvian Amazon, situated at 10 km from the main road (Iquitos-Nauta road) and 21 km southeast of Iquitos city (six hours by boat via the Itaya river) [14] (Figure 1). Malaria transmission is perennial with a peak during the rainy season (November–May). About 80% of malaria cases are caused by *P. vivax,* and *Anopheles darlingi* is the main vector [7,16]. The 127 habitants of San Carlos (2008 census) were primarily ‘mestizos’ (individuals not belonging to a specific ethnic minority) living in open wooden houses (average of 4.7 persons/house), mainly occupied in slash and burn agriculture and small scale fishery [17]. Households are situated close (13–45 m) to each other with the maximal distance between any two households being of less than 1 km (V Soto-Calle, pers comm). Neither electricity nor basic sanitation services are available, and the closest health post was approximately 3 km away from San Carlos community.

### Sample collection

*Plasmodium vivax*-infected participants were identified by microscopy examination of blood smears collected during an initial screening of the village population (April 2008) followed by active case detection of all fever cases (April to December 2008), following the study protocol of the larger cohort study. All microscopically confirmed *P. vivax*-infected participants were treated (directly observed treatment) with CQ 25 mg/kg/day (three days) and PQ 0.50 mg/kg/day (seven days), following the national guidelines for radical cure [5]. The most important limitation for the seven-day regimen in the Peruvian Amazon is the adherence to the treatment [18]. For this reason, the treatment was administered in a supervised way by a health care worker (hired exclusively for this study) and monitored every day during the seven-day treatment. Participants were asked their written informed consent (parents/guardians for minors) to be monthly follow-up for two years in order to detect *P. vivax* recurrent infections. Study subjects were visited at home weekly for the first month after treatment, and then monthly for the next 23 months. If a participant missed one scheduled sampling, the health care worker came back to the community two more times to look for the participant and/or scheduled the most feasible time to visit. At each visit, the medical staff asked study participants about any symptoms they may have had since the previous visit. A blood sample for microscopy (thick and thin film) and a blood spot on filter paper (BSFP) (Whatman grade 3, Whatman, Springfield Mill, USA) for later molecular analysis were systematically collected. Between scheduled visits, participants were encouraged to consult the study team whenever they felt ill, to check for fever and malaria infection (blood smear and BSFP). If malaria parasites were identified by microscopy in the field after recruitment, participants were treated with CQ for three days under direct supervision, and followed up until parasite clearance (confirmed by microscopy). Those participants with 2 or more episodes of malaria after the recruitment were given a complete 7-day PQ treatment before the 2 year follow up. This study was approved by the Ethical Boards of Universidad Peruana Cayetano Heredia, Peru (Project PVIVAX-UPCH, SIDISI code: 053256), the Institute of Tropical Medicine Antwerp and the University Hospital of Antwerp (Belgium).

### Laboratory methods

All day 0 (D0) blood samples were analysed by microscopy (thin and thick film) and *Plasmodium* species-specific PCR (ssPCR) to confirm the presence and density of *P. vivax* parasites, and this was repeated for all blood samples taken during participants’ monthly follow-up (scheduled and unscheduled visits). Parasite density was computed after counting the number of asexual parasites for 200 white blood cells (WBC) in the thick smear and assuming a concentration of 8,000 WBCs/μl. A slide was declared negative if no malaria parasite was found after examining 100 fields. Quality control was done by a senior technician on all positive and 10% of negative slides [19].

Parasite DNA was extracted from the BSFP using the saponin lysis Chelex 100 method [20], and either stored at 4°C for immediate use in PCR reactions or at -20°C for later use. All samples were first analysed by ssPCR following a protocol published elsewhere [21]. Briefly, this ssPCR consisted of a primary PCR with primers directed to specific *Plasmodium* and mammal sequences, and obtained amplicons were used for a second PCR using *Plasmodium*-species specific primers.

Positive vivax malaria samples, either by microscopy and/or species specific PCR, were selected for genotyping using 15 neutral, or nearly neutral, microsatellite markers (MS) as described elsewhere [22,23]. This technique consists of PCR amplification of 15 microsatellite sequences using for each reaction two specific pairs of primers (flurophore-labelled forward primer and no labelled reverse primer). The PCR fragments were analysed by capillary electrophoresis in a 3730 XL ABI sequencer (Applied Biosystems, Foster City, CA, USA). The size of the PCR fragments was determined using Genemapper (Applied Biosystems, Foster City, CA, USA); bands smaller than 100 relative fluorescence units (RFU) were defined as background. Samples presenting no microsatellite PCR (MS PCR) amplification in some loci were re-analysed [23].

### Data analysis

Data were double entered and cleaned in Excel (Microsoft, USA) and then analysed with SPSS v.16 (SPSS Inc, Chicago, IL, USA). A *P. vivax* recurrence was defined as any symptomatic (i e, fever: axillary temperature >37.5°C) or asymptomatic *P. vivax* infection identified by microscopy and/or PCR occurring between seven days and 24 months after the start of radical cure treatment. Recurrences were defined as “patent” if identified by microscopy, and “sub-patent” if detected only by ssPCR. All sub-patent infections were assumed to have an average parasite density of 10/μl [24]. The overall mean parasite density (geometric means) at day zero (D0) and overall density at monthly follow-up visits were compared by Student’s *t*-test.

### Genetic structure and population dynamics

An infection was defined as polyclonal (presence of two or more genetically distinct clones) if at least one locus presented more than one allele [25]. The percentage of polyclonal samples detected by each locus was described. The locus with the highest number of alleles was considered as a proxy for the ‘multiplicity of infection’ (MOI), representing the minimal number of parasite haplotypes in the sample [14]. Haplotypes, described as unique allelic combinations of the 15 loci analysed, were determined in monoclonal samples using Genclone v2.0 [26]. The haplotypes within polyclonal samples were analysed using both the predominant and minor alleles in the sample (peak size in the electropherogram) and defined respectively as the most probable dominant and minor clones. The proportions of monoclonal/polyclonal infections in children (≤14 years old) and adults (>14 years old) at D0 and among recurrent infections were compared using Pearson chi-square test.

The total number of alleles per each locus, the allelic richness (number of alleles per locus independently of the sample size), the number of alleles detected in a sample by any locus, and the genetic diversity of each locus (expected heterozygosity (*He*)) were computed for each population using FSTAT v.2.9.3 [27]. The *He* represents the probability of finding a different allele for a given locus in any pair of haploids randomly drawn from the same population. Genepop v4.0 software was used to assess the population differentiation through the analysis of the allele distribution [28,29].

The standardized index of association (*I*_*A*_^*s*^) was computed with LIAN 3.5 software [30] and used to test whether multilocus linkage disequilibrium (multilocus LD) occurred in local parasite population [11,12,14]. The *I*_*A*_^*s*^ was assessed among monoclonal infections, respectively for D0, monthly recurrences and all samples together. In addition, pair-wise linkage disequilibrium (pair-wise LD) was assessed between every pair of markers using G-statistics with FSTAT software [27] to avoid any bias due to physical linkage between particular loci located within the same contigs (relatively long segments of contiguous DNA sequence assembled during genome analysis) [11]. The probability of finding identical haplotypes derived from distinct sexual reproductive events (*p*_*sex*_) in two or more samples was computed using GenClone ver. 2.0 [26].

Three different approaches (eBURST, STRUCTURE and NETWORK) were used to identify clonal parasite groups. eBURST software v.3 [31] was used to identify *haplogroups*, defined as clusters of closely related haplotypes that are identical to each other on at least 11 loci [13]. The haplotypes that were not related to any haplogroup were classified as *singletons.* The haplogroups and singletons were used to analyse the transmission dynamics of the parasites at the genetic level over time [11,13]. In addition, STRUCTURE v.2.3.3 was used to identify distinct sub-populations and determine fractions of the haplotype for each strain that belong to each sub-population. The most probable number of clusters was defined by calculating the K value as described elsewhere [32]. The relationship between *P. vivax* haplotypes was further analysed by a simple phylogenetic approach using software Network v.4.6.1.0 [33].

Using the geo-coding of the participants at household level and the SaTScan software v9.1.1 [34], space-time scan statistics were employed to identify clusters of space-time malaria cases with elevated proportions of specific haplotypes. This was done by gradually scanning a window across time and space, noting the number of observed and expected infections inside the window at each location. The space-time clusters were assessed with up to a maximum of 50% of the data included in the scanning window and a maximum temporal window size of up to 50% of the study period. The relative risk (RR) was computed by dividing the estimated risk within the cluster by the estimated risk outside the cluster. The window with the maximum likelihood is defined as the most likely cluster. The significance of the identified space-time clusters was tested using the likelihood ratio test statistic and *p-values* were obtained through Monte Carlo simulations [35].

## Results

### Baseline characteristics

**Figure 2:**
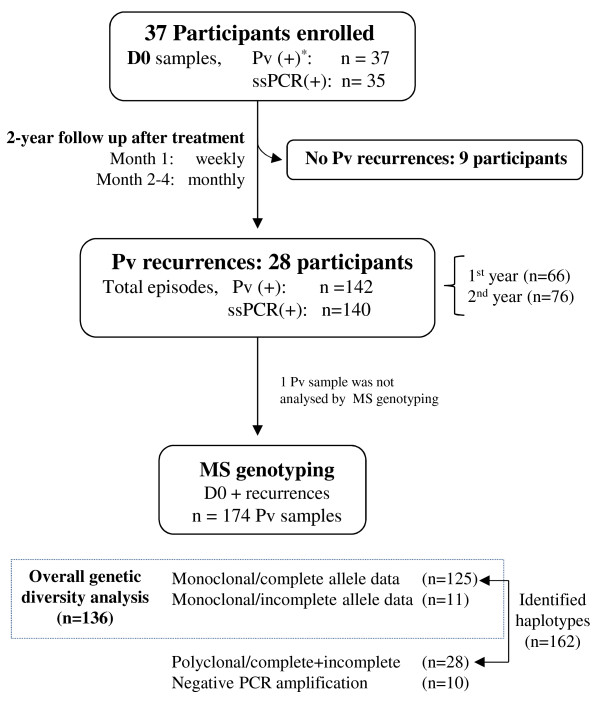
**Study flowchart.** Thirty-seven participants were enrolled and treated (CQ + PQ) on D0. Twenty-eight participants had at least one recurrence and nine participants had no recurrences after treatment. Only samples positive for *P. vivax* by ssPCR were genotyped with 15 microsatellites (MS). In total, 162 haplotypes were detected in monoclonal and polyclonal *P. vivax* samples. From 28 polyclonal infections only 37 haplotypes were identified as described in the Methods section. * = microscopically positive. Pv = *P. vivax.*

In April 2008 (screening), the prevalence of vivax malaria in San Carlos was estimated at 10.2% by standard microscopy. Between April and December 2008, a total of 37 individuals (in 20 households) were identified with a *P. vivax* mono-infection and recruited into the study. Males (n = 19) and females (n = 18) were equally represented, and the median age was 15 years (IQR 3–48) with 40% of study participants aged < nine years. At D0 the mean parasite density was 2,031/μl (95% CI: 1,332-3,098). Though all D0 slides were double-read by a senior technician, the ssPCR remained negative (ssPCR repeated once after negative amplification) for two participants whose parasite density by microscopy was 48/μl and 823/μl, respectively. During the first 28 days of follow-up, there was no *P. vivax* recurrence observed by microscopy or by ssPCR (Figure 2).

### *Plasmodium vivax* recurrences during the monthly follow-up

At day 28, all 37 participants were found negative both by microscopy and ssPCR. During the following 23-month period, a total of 142 *P. vivax* recurrent infections were detected by microscopy with a mean parasite density of 88/μl (95% CI: 56–139) which was significantly lower than that at D0 (p < 0.001). The ssPCR missed two recurrent infections despite a parasite density by microscopy of 36 and 4,352/μl, respectively. By the end of the two-year follow-up, 76% (28/37) of the study subjects had experienced at least one recurrent *P. vivax* infection, while only two *Plasmodium falciparum* infections were identified. More than half of the recurrent infections (54.9%, 78/142) were sub-patent and asymptomatic.

### Genetic diversity of the *Plasmodium vivax* population

A total of 174 *P. vivax* infections (35 D0 and 139 recurrences) were genotyped by MS PCR (Figure 2), among which 164 had a MS PCR amplification. Most of them (82.9%, 136/164) were monoclonal and had complete allele data (for all loci) (91.9%, 125/136). The proportions of monoclonal and polyclonal infections did not differ between baseline and recurrent infections, and neither did the average multiplicity of infection (MOI) (Table 1). The average expected heterozygosity (*He*) was 0.49 (range 0.10-0.67) (Table 1), and MS4 was the most informative marker (*He* = 0.65) discriminating 58.6% of all polyclonal infections. The average total number of alleles per locus was 4.6 (range 2–7), the average allelic richness was 4.17 (range 1.99-6.28) and the average number of alleles detected in a sample by any locus remained constant between all loci (1.1-1.2). Average genetic indexes did not vary between D0 and recurrences, however looking at specific periods of the study, differences were found, as shown later.

**Table 1:** **Overall polymorphism of the 15 *Plasmodium vivax *** **microsatellite loci assessed in San Carlos community**

**Locus**	**Total number of alleles**	**Allele size (range in bp)**	***He***	**Allelic richness**	**Polyclonal samples/locus (%)**	**Average alleles/locus**
MS1	4	234 - 242	0.1	2.94	0%	1.1
MS2	5	187 - 207	0.6	4.89	13.8%	1.1
MS3	2	181 - 184	0.13	1.99	6.9%	1.1
MS4	5	165 - 198	0.65	4.62	58.6%	1.2
MS5	2	166 - 172	0.29	2	6.9%	1.1
MS6	5	208 - 244	0.61	4.38	17.2%	1.1
MS7	3	142 - 148	0.47	2.98	17.2%	1.1
MS8	4	252 - 271	0.6	4	24.1%	1.1
MS9	5	155 - 172	0.66	4.94	20.7%	1.1
MS10	4	210 - 266	0.13	3.19	0%	1.1
MS12	6	181 - 332	0.55	5.14	27.6%	1.1
MS15	4	210 - 256	0.59	3.98	31%	1.1
MS16	7	193 - 350	0.67	5.92	13.8%	1.1
MS20	7	194 - 241	0.65	6.28	24.1%	1.1
Pv6635	6	184 - 202	0.67	5.34	27.6%	1.1

A total of 174 *P. vivax* infections were analysed, including 35 D0 and 139 recurrent samples. The expected heterozygosity (*He)* and the allelic richness were calculated only for monoclonal samples (n = 136).

### Parasite inbreeding

**Figure 3:**
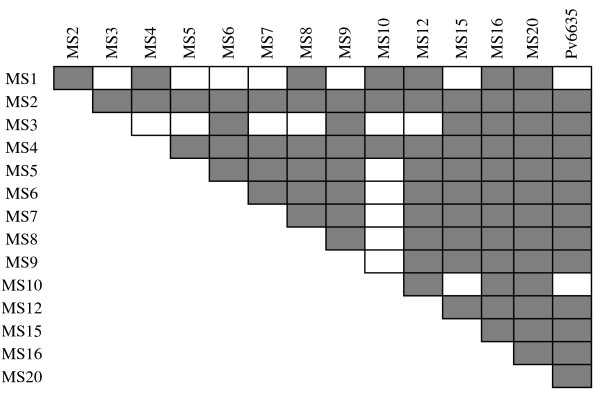
**Patterns of linkage disequilibrium between pairs of microsatellite markers in the *Plasmodium vivax *population from San Carlos community.** (n = 136 monoclonal samples analysed). Grey shading denotes linkage disequilibrium at the 5% significance level (adjusted p =0.00005 based on 10^6^ permutations).

The analysis of the monoclonal samples (n = 136) showed a highly significant overall LD (*I*_*A*_^*s*^ = 0.51, p < 0.0001) indicating a clonal population. To check for bias due to unequal distribution of the haplotypes or due to sampling, the LD was also assessed for unique haplotypes only, and it remained significant (*I*_*A*_^*s*^ = 0.15, p < 0.0001). Inbreeding, rather than physical linkage between markers established in the same contig (MS4-MS5, MS7-MS8 and MS12-MS15), was identified by the linkage between multiple loci obtained by pair-wise LD analysis (Figure 3). Indeed, the probability that the clonal population was due to reproductive events between genetically unrelated parents was very low (*p*_*sex*_ = 0.0006).

### *Plasmodium vivax* population structure

**Figure 4:**
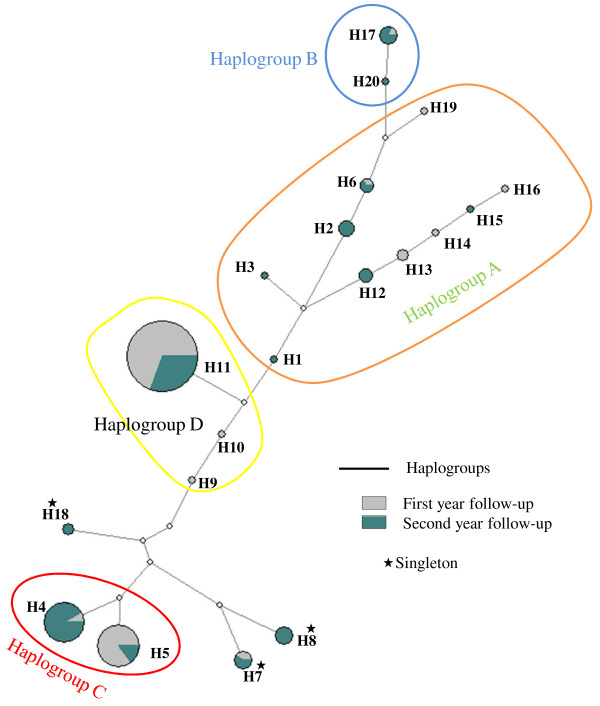
**A median joining tree performed using NETWORK representing the relationship between *Plasmodium vivax *haplotypes and their temporal distribution.** Each circle represents a different haplotype and the size reflects the frequency of detection within the two years (n = 162). Related haplotypes are linked by a line and mutations (о). Solid lines denote the haplogroups obtained using eBURST v.3 and STRUCTURE v.2.3.3 (Pop A, B, C, D).

A total of 162 haplotypes were identified, 125 from monoclonal and 37 from polyclonal infections, of which 20 were classified as unique haplotypes (Figures 2 and 4). H11, H4 and H5 were the most frequent haplotypes detected in 45, 15 and 16% of the infections, respectively. Moreover, eight haplotypes were detected two to five times (<3% each) and nine haplotypes were detected only once. Using eBURST (criteria = 11 common loci), four haplogroups (A, B, C, and D) containing two to ten unique haplotypes and three singletons were identified. Similarly to eBURST, four populations (k = 4) were inferred using STRUCTURE and were very similar to the haplogroups previously defined by eBURST (Figure 4 and Additional file 1). The phylogenetic tree obtained using NETWORK supported the classification results obtained by eBURST: i) haplogroup A contained ten low frequency, slightly different haplotypes; ii) haplogroup B, derived from haplogroup A, contained only two haplotypes (two extra haplotypes, H8 and H18, considered as singletons by eBURST belonged to this group following the classification by STRUCTURE (Pop. B); and, iii) and iv) haplogroup C and D contained two to three haplotypes but were dominant throughout the study (Figure 4).

### Parasite dynamics and space-time clustering

**Figure 5:**
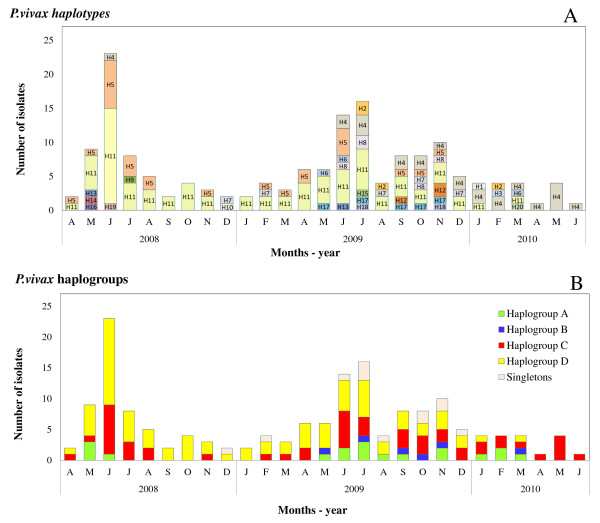
**Transmission dynamics of *Plasmodium vivax *haplotypes and haplogroups in San Carlos community.***Panel****A****:* Monthly distribution of the 162 *P. vivax* haplotypes found among 164 *P. vivax* infections (patent and sub-patent) between April 2008 and June 2010. Twenty unique haplotypes were detected and are represented by different colours. *Panel****B****:* Monthly distribution of the haplogroups containing closely related haplotypes determined using eBURST. Composition of each haplogroup: A (H1, H2, H3, H6, H12, H13, H14, H15, H16, H19), B (H17, H20), C (H4, H5), D (H9, H10, H11), singletons (H7, H8, H18).

The genetic diversity (*He)* did not vary substantially between baseline (D0) and first year monthly follow-up samples, 0.44 and 0.40 (p = 0.26), respectively. Nevertheless, *He* was significantly higher during the second year of follow-up (*He* = 0.51) compared to both baseline and first year (p < 0.0001). In addition, compared to the first year, multilocus LD (*I*_*A*_^*s*^) during the second year decreased from 0.6 to 0.4. The frequency of the most common haplotype at D0 and during the first year, i e, H11 (=haplogroup D), decreased substantially during the second year (from 60 to 30%, p < 0.01), while in the same period new infrequent haplotypes (i e, H1, H3) and other haplotypes belonging to the haplogroup A were observed (Figure 5, panel A). The frequency of the haplogroup C remained constant throughout the study although its composition varied by year, H4 gradually replacing H5 during the second year (Figure 5, panel B).

**Figure 6:**
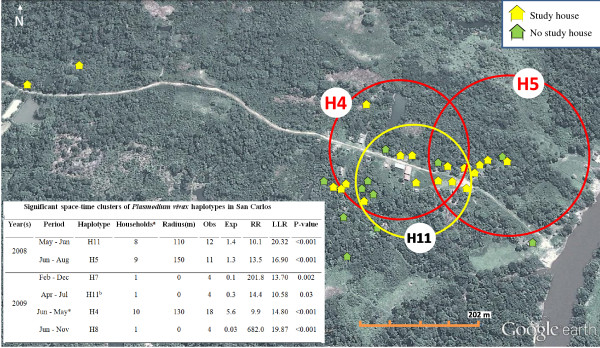
**Aerial view of San Carlos depicting the location of the three major space-time primary clusters of *Plasmodium vivax *haplotypes (H11, H5 and H4) and a description of all the significant clusters found during the follow up.** Households mapped with GPS on Google Maps. ^a^ Number of households by cluster; ^b^ Secondary cluster; Obs = observed number of cases; Exp = expected number of cases; RR = relative risk; LLR = log likelihood ratio. Radius in meters (m). *H4 cluster from June 2009 until May 2010.

Using SaTScan, a total of six space-time clusters of five different haplotypes (out of 20) were identified during the study period (Figure 6). Clusters of H11 and H5 represented the two main clusters within the first year (RR = 10.1 and 13.5 respectively; from May to August 2008), while H4 cluster represented a significant large cluster during the second year (RR = 9.9; from June 2009 to May 2010). In 2009, H11 was defined as a secondary cluster, and together with two other haplotypes (H7, H8) clustered in single households for several months. Due to sparseness of some haplotypes in multiple households, only clusters with meaningful RR (not zero nor infinity) were reported.

### Dynamics at individual level

More than two thirds of the participants (68.4%, 26/37) had infections with one to two different haplotypes during their follow-up period, while the remaining had infections with three to five haplotypes (up to three different haplogroups or singletons). The most frequently detected haplotype, H11, dispersed within 80% (16/20) of the participants’ households and occurred in 60.7% (17/28) of the participants in at least one infection (up to eight infections with H11 in two participants).

One or more sub-patent recurrent infections that cleared without treatment were observed in 50% (14/28) study subjects (see Additional file 2). Some of these infections had the same haplotype over several consecutive months. During their follow-up, 60%, (9/15) of study subjects with a H11 haplotype infection at recruitment had several sub-patent recurrent infections with the same haplotype.

## Discussion

In San Carlos, a relatively isolated village in the Peruvian Amazon, the majority (76%) of the 37 study participants experienced *P. vivax* recurrences after radical cure given according to the national guidelines. Most of the *P. vivax* recurrences were sub-patent and asymptomatic, some of them persisting for several months. Such a high occurrence of recurrent infections after treatment with a full course of PQ has already been observed in several other South American endemic countries [13,36-39]. The large proportion of monoclonal *P. vivax* infection found in this study was similar to that reported from Colombia [40] but different compared to other communities in the Peruvian Amazon [14] and other areas in South and Central America [11,12]. This is probably due to the differences in effective recombination rates and in human and vector behaviour. The clonality of the parasite population in San Carlos was confirmed by the highly significant LD. The latter can be due to either the low rate of recombination (inbreeding) or the recent occurrence of admixture of two unrelated genotypes that did not have sufficient time to homogenize [41]. Though the latter is theoretically possible, considering the strong differentiation between malaria parasite populations over relatively small geographical distances reported in South America [11,42,43], the low probability of admixture (*p*_*sex*_) found in San Carlos indicates that inbreeding was the most likely reproductive event in this area. This was also the case in other reports from endemic areas of the Amazon Basin [11,13,43]. Inbreeding could also explain the replacement of haplotypes without haplogroup replacement as it was observed in this study with the highly prevalent haplogroup C.

Clustering of malaria transmission at household level has been previously reported in the Peruvian Amazon [6,14]. In the present study, malaria infections carrying the same haplotype, or haplotypes from the same haplogroup, were identified concurrently or within a period of a few weeks in participants living in the same households. The persistence of infections with the same haplotype for long periods, also due to the relapsing nature of *P. vivax*[44,45], can maintain a certain level of immunity even in low transmission areas like the Amazon Basin [46].

The clustering of haplotypes may also influence the parasite diversity and the interpretation of recurrent parasitaemia. Some participants carried the same haplotype for several consecutive months, and later presented a new but closely related haplotype (Additional file 2). Previous studies have reported also a high rate of genetically similar recurrent infections in the Peruvian Amazon [44,47]. It was not possible to determine whether this genetic variation originated within the mosquito or the human host. Nevertheless, considering the low transmission in San Carlos, it is likely that most recurrences were not new infections, but rather relapses or recrudescences. For example, haplotypes H4 and H5 (distributed in 8/20 and 14/20 households, respectively) infected participants living within the same households (in seven households in total). H4 and H5 belong to the same haplogroup and they differ by only one locus (MS8, alleles of 258 and 271 bp, respectively). As most of the *P. vivax* microsatellite diversity is produced within the human host during the mitotic replication [48-50] and considering that H4 displaced H5 in the second year, deletion of repeat units may have caused the haplotype differentiation in the human host. If this is the case, the recurrence of heterologous parasites may not be necessarily due to previous infection(s) with multiple different clones [51] but to genetic variation of one parasite clone within the human host. In addition, homologous parasites in recurrent infections may represent relapses of a limited diversified parasite population or non-hypnozoite stages (i.e., parasite stages within splenic dendritic cells) that re-activate after extended periods of latency [5,52,53].

### Clonal population: possible implications for malaria epidemiology

Due to the clonality and clustering of the parasite population in San Carlos, people were repeatedly exposed to the same strains. According to the model of strain-specific immunity to malaria [10,54], effective malaria immunity in low transmission areas may be achieved after a relatively small number of infective mosquito bites [41]. In addition, in areas with low levels of recombination, haplotypes may be maintained through multiple generations [41], continuously exposing their antigens to the human immune system [2]. In San Carlos, most participants were asymptomatic to the most frequent haplotype, H11, possibly due to the development of clinical immunity that was able to control the parasite density and prevent symptoms. Therefore, the limited genetic diversity and the low MOI in this low transmission area may still stimulate an efficient immune response [10] which could explain the common occurrence of asymptomatic and sub-patent infections.

In relatively isolated populations such as San Carlos, with few circulating clones, continued anti-malaria treatment/drug pressure may foster a genetic restructuring of the parasite population, favouring the spread of the drug resistance trait [9]. No indication of CQ resistance was found in the present study. However the frequent occurrence of recurrent infections with similar haplotypes after one month the radical treatment was given could be an early sign that the PQ is not working efficiently, probably due to resistant parasites or inefficient metabolic activation of the PQ by the participants. Further studies involving a larger number of endemic communities, with different rates of recurrences and parasite population structure, as well as the use of *in vitro* studies, resistance markers and whole parasite genome sequencing would give more insights on the rise and spread of resistant parasites and the parasitic genetic diversity.

## Conclusions

Combining population genetic tools and space-time scan statistics allowed for an in-depth insight to the dynamics of *P. vivax* clones at individual and household level in this isolated community of the Peruvian Amazon. Space-time clustering of recurrent asymptomatic *P. vivax* infections with few specific clones reflects the highly focal transmission of the local parasite population favouring the development of clinical immunity by infected people. This type of setting could also be at high risk of selection and spread if drug resistant *P. vivax* clones are under high selective pressure. This type of information is highly valuable for further strengthening malaria elimination strategies in the Peruvian Amazon and should be extended to different endemic areas.

## Competing interests

The authors declare that they have no competing interests.

## Authors’ contributions

Conceived and designed the experiments: CD, VESC, PVdE, DG, HRF, ALC, AE, UDA. Performed the experiments: CD. Analysed the data: CD, PVdE, AR, AE, UDA. Contributed reagents/materials/ analysis tools: VESC, AR, ENA, DG, AR, AE. Wrote the paper: CD, VESC, DG, JPVG, AE, UDA. All authors read and approved the final manuscript.

## Supplementary Material

Additional file 1 **Population structure inferred by microsatellite genotyping of 136 *Plasmodium vivax *** **monoclonal infections using STRUCTURE.***Panel A,* using the method described elsewhere [32]*,* the uppermost hierarchical level of structure was assumed, describing four populations (highest peak, K = 4). Two other peaks (K = 6 and K = 9) are described with a lower likelihood. K = 6 corresponded to the six haplogroups defined by eBURST when the criteria of relatedness was increased to 14 loci instead of 11. *Panel B* illustrates the bar plot at K = 4 with 136 samples being represented by a single vertical line divided into colours assigned to the population of origin. Each colour represents one population (A-green, B-blue, C-red, D-yellow), and the length of the coloured segment shows the estimated proportion of membership of that sample to each population. Click here for file

Additional file 2 **Characteristics of each malaria infection suffered by the 37 participants.** X = Initial infection in this study (D0). Green shading indicates a recurrent *P. vivax* infection. P = patent infection. On the upper left corner of the cells is defined the haplotypes (H1…H20) or one of the symbols explained below. *i* = incomplete allele data, *m* = mixed infections (not possible to differentiate the haplotypes). *ind =* haplotype within a mixed infection that cannot be determined. *n* = *P. vivax* sample that did not amplified by PCR with any of the microsatellites. ^†^ Patent infection detected by microscopy but no by ssPCR (MS genotyping was not done). ^‡^ Pv positive by microscopy on D0 but filter paper D0 was not available. On day 1 was negative by ssPCR (MS genotyping was not performed). Click here for file
