# Supplementary figures and images for: Population structure and spatio-temporal transmission dynamics of Plasmodium vivax after radical cure treatment in a rural village of the Peruvian Amazon
Source: Malar J. 2014 Jan 6;13:8. doi: 10.1186/1475-2875-13-8 (PMC3893378; doi:10.1186/1475-2875-13-8)

# K populations (structure)

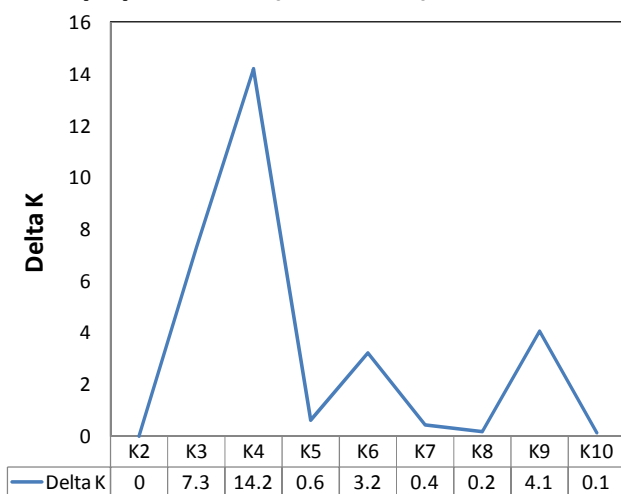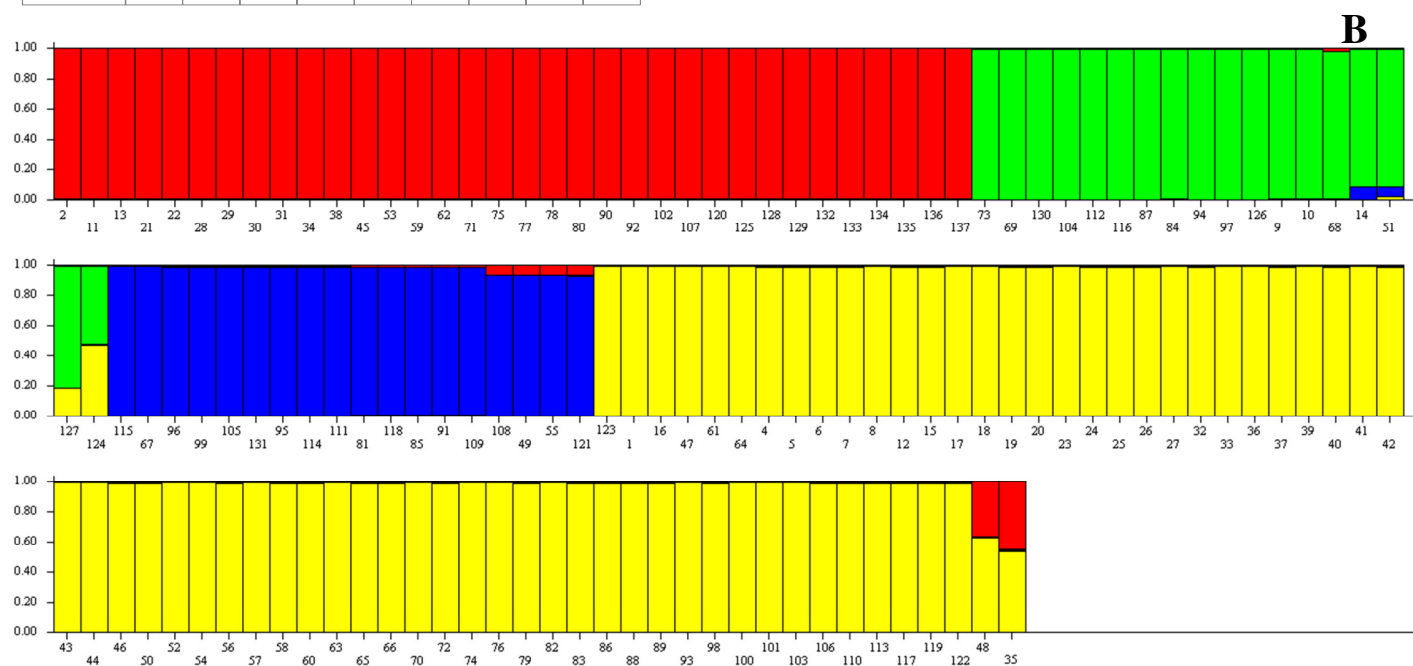

Supplement: Additional file 1 — Population structure inferred by microsatellite genotyping of 136 Plasmodium vivax monoclonal infections using STRUCTURE.Panel A, using the method described elsewhere [32], the uppermost hierarchical level of structure was assumed, describing four populations (highest peak, K = 4). Two other peaks (K = 6 and K = 9) are described with a lower likelihood. K = 6 corresponded to the six haplogroups defined by eBURST when the criteria of relatedness was increased to 14 loci instead of 11. Panel B illustrates the bar plot at K = 4 with 136 samples being represented by a single vertical line divided into colours assigned to the population of origin. Each colour represents one population (A-green, B-blue, C-red, D-yellow), and the length of the coloured segment shows the estimated proportion of membership of that sample to each population. [file 1475-2875-13-8-S1.pdf]
