# Supplementary material for: Population structure and spatio-temporal transmission dynamics of Plasmodium vivax after radical cure treatment in a rural village of the Peruvian Amazon
Source: Malar J. 2014 Jan 6;13:8. doi: 10.1186/1475-2875-13-8 (PMC3893378; doi:10.1186/1475-2875-13-8)
Supplement: Additional file 2 — Characteristics of each malaria infection suffered by the 37 participants. X = Initial infection in this study (D0). Green shading indicates a recurrent P. vivax infection. P = patent infection. On the upper left corner of the cells is defined the haplotypes (H1…H20) or one of the symbols explained below. i = incomplete allele data, m = mixed infections (not possible to differentiate the haplotypes). ind = haplotype within a mixed infection that cannot be determined. n = P. vivax sample that did not amplified by PCR with any of the microsatellites. † Patent infection detected by microscopy but no by ssPCR (MS genotyping was not done). ‡ Pv positive by microscopy on D0 but filter paper D0 was not available. On day 1 was negative by ssPCR (MS genotyping was not performed). [file 1475-2875-13-8-S2.pdf]

Household	Participant	2008									2009												2010					
		Apr	May	Jun	Jul	Aug	Sep	Oct	Nov	Dec	Jan	Feb	Mar	Abr	May	Jun	Jul	Aug	Sep	Oct	Nov	Dec	Jan	Feb	Mar	Abr	May	Jun
I	001				H5 X				H5								H18 **P**				H18 P							
II	002			H5 ind X						n	n			n			H8 **P**											
	003			H5 H11 X		n		n	H11	n	H11	H11	H11		n				H11									
	004									H7 X						H5 **P**	H11 P				H11		H11 H4 P					
III	005				H5 X					n				H11	H11						i		m P		m P			H4 P
IV	006			H5 **X**																								
	007			H11 **X**				H11 P																				
	008			H5 H11 **X**										H5 **P**		H13 **P**	H11				H12 P		H1 **P***					
	009			H11 **X**					H11 P						H17 P		H17 P		H17	H17	H17 P	† P			H20 **P**			
V	010			† X															H11	H11		H11 P		H3 P				
VI	011			H5 X																								
VII	012		H11 **X**				H11 **P**							H11	H11	H11	H11	H11		H11								
VIII	013	H5 X		H11 P												H5						H4						
IX	014		H13 ind X		H5	H5 P									i	H4 ind P			H12		H12		H4 **P**		H4	H4	H4	
	015			H11 **X**		H11 P		n								H5 P	H4 P										H4 **P**	
X	016			H11 X																								
	017			H11 **X**																								
	018			H11 X				H11 P							H6 **P**		H4 ind P		H4	H4		H4 ind		H2	H6		H4 ind	
	019			H11 **X**		H11 P	H11	† P			H11	H11		H11	H11	H11	H2 P											
XI	020			M **X**																								
	021			H11 **X**								H5 P																
	022			H11 **X**																								
XII	023			H11 X												H6 H11 **P**												
	024			H5 **X**									n															
XIII	025	ǂ X			H11 P							H7 P		H11	H11	H11	H11	H7 H11 P		H7		H7 **P**						
XIV	026		H5 X																									
XV	027		H11 X			H11					i						H15				H11				H11 ind			
	028		H11 X																									
	029		H11 X		H11 ind **P**			H11 ind P								H11												
XVI	030		H11 X		H11 ind P											H8	H8			H8	H8							
XVII	031	H11 X																										
XVIII	032		H14 X	H5 **P**		H5 ind **P**							H5 ind	m≠	m≠ **P**		H4							H4			H4	
	033		H16 X										H11 ind P		m **P**		H11 P		m		H11	H11 P						
XIX	034				H11 X											H5			H4 H5	H4 H5								
	035			H11 X				H11											H11									
XX	036			H4 **X**	H9 P					H10 **P**						H4 **P**		H2 **P**			H4 H5 P			H4 **P**				
	037			H19 **X**													H2 **P**											
